# Direct effects of elevated dissolved CO_2_ can alter the life history of freshwater zooplankton

**DOI:** 10.1038/s41598-022-10094-2

**Published:** 2022-04-12

**Authors:** Lana Ramaekers, Tom Pinceel, Luc Brendonck, Bram Vanschoenwinkel

**Affiliations:** 1 Community Ecology Laboratory, Department of Biology, Vrije Universiteit Brussel (VUB), Pleinlaan 2, 1050 Brussels, Belgium; 2 Animal Ecology, Global Change and Sustainable Development, KU Leuven, Ch. Deberiotstraat 32, 3000 Leuven, Belgium; 3 Centre for Environmental Management, University of the Free State, P.O. Box 339, Bloemfontein, 9300 South Africa; 4 Water Research Group, Unit for Environmental Sciences and Management, North-West University, Private Bag X6001, Potchefstroom, 2520 South Africa

**Keywords:** Climate-change ecology, Freshwater ecology, Limnology, Climate-change impacts

## Abstract

Dissolved CO_2_ levels (pCO_2_) are increasing in lentic freshwaters across the globe. Recent studies have shown that this will impact the nutritional quality of phytoplankton as primary producers. However, the extent to which freshwater zooplankton may also be directly affected remains unclear. We test this in three model species representative of the main functional groups of primary consumers in freshwaters; the water flea *Daphnia magna*, the seed shrimp *Heterocypris incongruens* and the rotifer *Brachionus calyciflorus*. We experimentally exposed individuals to three pCO_2_ levels (1,500; 25,500 and 83,000 ppm) to monitor changes in life history in response to current, elevated and extreme future pCO_2_ conditions in ponds and shallow lakes. All species had reduced survival under the extreme pCO_2_ treatment, but the water flea was most sensitive. Body size and reproduction were reduced at 25,500 ppm in the water flea and the seed shrimp and population growth was delayed in the rotifer. Overall, our results show that direct effects of pCO_2_ could impact the population dynamics of freshwater zooplankton. By differentially modulating the life history of functional groups of primary consumers, elevated pCO_2_ has the potential to change the evolutionary trajectories of populations as well as the ecological functioning of freshwater communities.

## Introduction

Atmospheric carbon dioxide levels currently reach ~ 410 ppm, which is an increase of > 40% since 1750^1,2^ and levels are predicted to further increase up to ~ 900 ppm by 2100 under the RCP8.5 scenario^3^. This translates to increased CO_2_ levels (pCO_2_) and accompanied acidification in aquatic ecosystems across the globe^4–6^. Most freshwaters are characterized by substantial seasonal and daily fluctuations in CO_2_ levels^7,8^ and as a result of high soil respiration and net heterotrophy, they are often already supersaturated^9,10^. Concentrations currently range between 36 and 23,000 ppm in global freshwaters^11^, but extreme values up to 60,000 and 80,000 ppm have been reported^12,13^. Because of continued emission, changes in terrestrial primary productivity as a source of allochthonous carbon and high respiration rates, pCO_2_ levels will probably continue to increase^6^. Although this is expected to severely impact freshwater organisms, most studies have focused on potential effects on phytoplankton, while impacts at higher trophic levels are much less studied^14^.

A number of studies have shown that higher carbon availability in the form of CO_2_ will lead to higher primary production of phytoplankton^15,16^ and changes in community composition^17,18^. The resulting increase in the tissue carbon content relative to nitrogen and phosphorus can affect the nutritional value of phytoplankton as food for zooplankton as the main primary consumers^19,20^. Although freshwater zooplankton can thus be affected indirectly via their main food source, it remains unclear to what extent they may also respond directly to the higher abundance of CO_2_ molecules in the water or the associated acidification. Sublethal responses that have been linked to elevated pCO_2_ include changes in behaviour, calcification and ion regulation in freshwater bivalves and a crab species^21–23^. Urabe et al. (2003)^19^ exposed the water flea *Daphnia pulicaria* to an elevated pCO_2_ of 3,500 ppm while feeding algae grown under ambient pCO_2_ conditions (360 ppm) but found no effects on growth. In two different water flea species of the genus *Daphnia*, no effect of elevated pCO_2_ of 11,000 and 16,000 ppm on body size was detected although a reduction in the development of anti-predator defenses was found^6,24^.

An important limitation of this earlier work is that these were short term experiments (typically < 7 days) in which a limited number of life history traits was considered. To get a more reliable idea of the potential impact on population dynamics, longer experiments are needed and a wider range of life history traits should be considered. In addition, current work on the impact of pCO_2_ on primary consumers in freshwater has been largely restricted to water fleas of the genus *Daphnia*. It is, however, important to consider additional functional groups of primary consumers to get an idea to what extent they might be more or less sensitive since differential sensitivity may alter competitive relationships in ponds and shallow lakes.

The main goal of our study was to investigate to which extent elevated pCO_2_ and associated weak acidification can directly affect the life history of different primary consumers in freshwater ponds and lakes. Therefore, we selected model species that represent three main functional groups of primary consumers, the water flea *Daphnia magna* (large pelagic filter feeders), the seed shrimp *Heterocypris incongruens* (benthic collector gatherers/ medium-sized pelagic filter feeders) and the rotifer *Brachionus calyciflorus* (small pelagic filter feeders) (see also Appendix 3). Although most earlier studies used artificial culture medium, we used filtered natural pond water to better mimic the complex chemistry of ponds resulting in a realistic buffering capacity. By feeding standardized dead algae, we could analyze direct effects of pCO_2_ while preventing any indirect effects by altering food availability or quality^19^.

Our experiment was conducted under different pCO_2_ conditions, including an average current level (~ 1,500 ppm; C)^12^ as well as elevated (~ 25,500 ppm; T1) and extreme (~ 83,000 ppm; T2) concentrations. Although high, these concentrations are ecologically relevant as they reflect peak values that have already been measured today^11^ (Table S1, Appendix 1). In poorly buffered systems, the tested concentration of 25,500 ppm could become common considering indirect mechanisms that contribute to increasing pCO_2_ such as elevated terrestrial primary production^4,25^. In addition, longer peak periods are likely to become more common^6,26,27^. The extreme level coincides with the upper limit currently reported in freshwater^13^ and it could potentially occur more frequently under extreme future conditions. Extreme pCO_2_ levels might become more common in North American rivers as a result of invasive species management using CO_2_ gas as a barrier for fish^21,22,28^. However, we primarily included it as a test of the tolerance limits of extant species.

Overall, we aimed to (1) provide evidence for the direct effects of pCO_2_ on life history and survival of different functional groups of freshwater zooplankton as a proof-of-principle and (2) explore possible differential sensitivity of three model species that represent three functional groups of primary consumers that coexist in most of the world’s lentic freshwater ecosystems. We hypothesized that the 83,000 ppm pCO_2_ concentration would jeopardize survival since it exceeds typically reported values for freshwater systems^11,12^. We expected that the crustaceans would be most affected since they have a relatively calcium-rich exoskeleton and elevated pCO_2_ and reduced pH may interfere with calcification processes^22,23,29^. We expect effects to be particularly strong in the ostracod since they have highly calcified valves^30^. Secondly, we hypothesized that the physiological stress associated with high pCO_2_ and weak acidification would lead to reduced growth and reproduction due to increased energy investment in homeostasis^31,32^.

## Results

**Figure 1:**
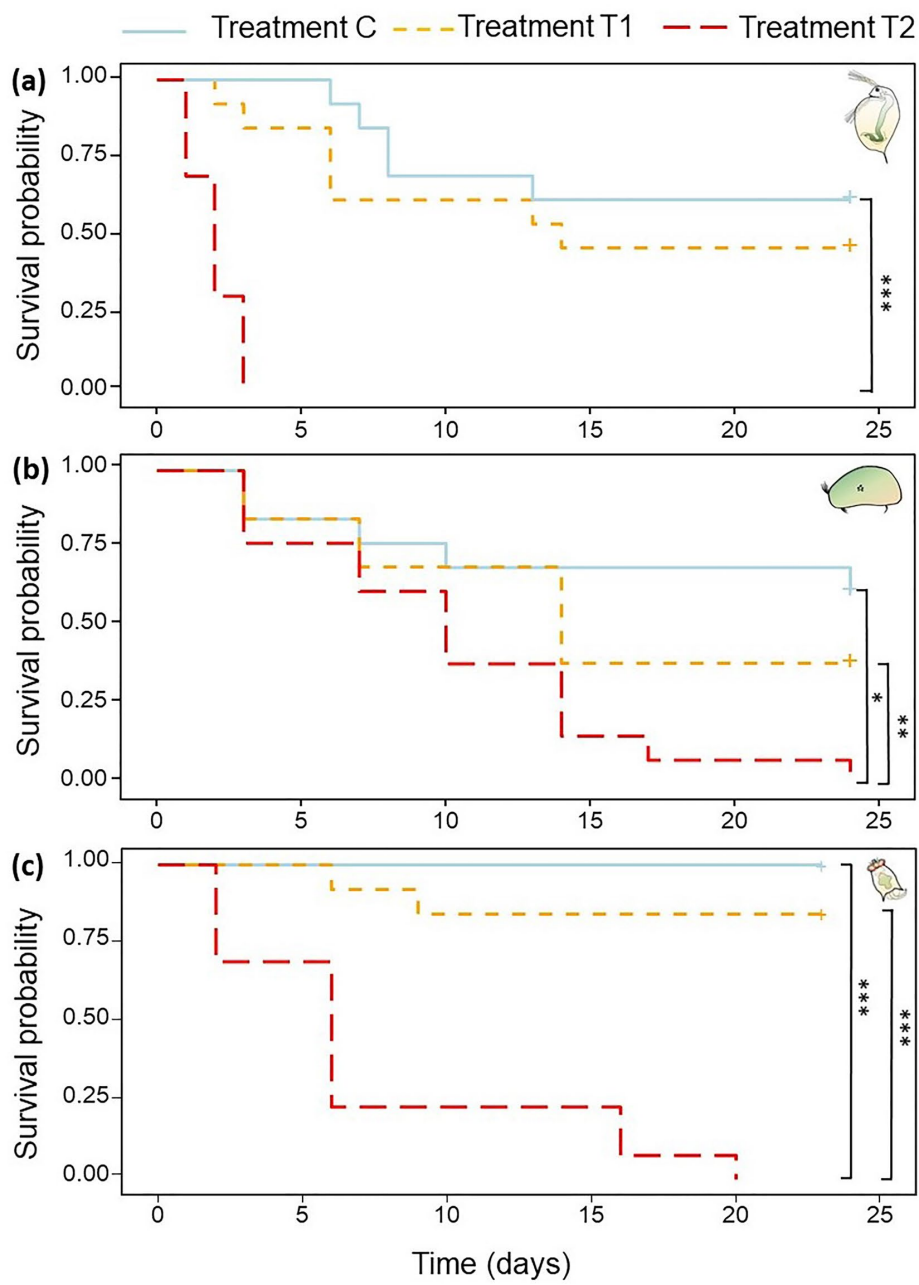
Kaplan–Meier curves showing survival probability over time of three freshwater zooplankton species subjected to a control (C = 1,520 ppm), an elevated (T1 = 25,609 ppm) and an extreme (T2 = 83,201 ppm) pCO_2_ treatment. Log-rank test results are shown for (**a**) individual survival of the water flea *D. magna*, (**b**) individual survival of the seed shrimp *H. incongruens* and (**c**) survival of clonal populations of the rotifer *B. calyciflorus*. Plus signs ( +) indicate right censored data. Significant differences are indicated with asterisks: *p < 0.05, **p < 0.01, ***p < 0.001.

pCO_2_ significantly reduced survival in all species, but only at the highest concentration (−100% in the water flea on day 3: Log rank test, χ^2^_2_ = 39.1, *p* < 0.001; −39% in the ostracod on day 24: Log rank test, χ^2^_2_ = 10.2, *p* = 0.006; −100% in the rotifer on day 20*:* Log rank test, χ^2^_2_ = 40.6, *p* < 0.001; Fig. 1). In this extreme pCO_2_ treatment, all water flea individuals died within 3 days while seed shrimp and rotifers survived longer (Log rank test, χ^2^_8_ = 132, *p* < 0.001; Pairwise comp., water flea-seed shrimp: *p* < 0.001, water flea flea-rotifer: *p* < 0.001), only reaching 100% mortality after 20 and 24 days, respectively. Rotifers and water flea survival rates were similar under T1 and higher than those of seed shrimp under T1 (Pairwise comp., rotifer-seed shrimp: *p* = 0.043). Survival under T1 differed between water flea clones (Log rank test, χ^2^_4_ = 15.3, *p* = 0.004).

**Figure 2:**
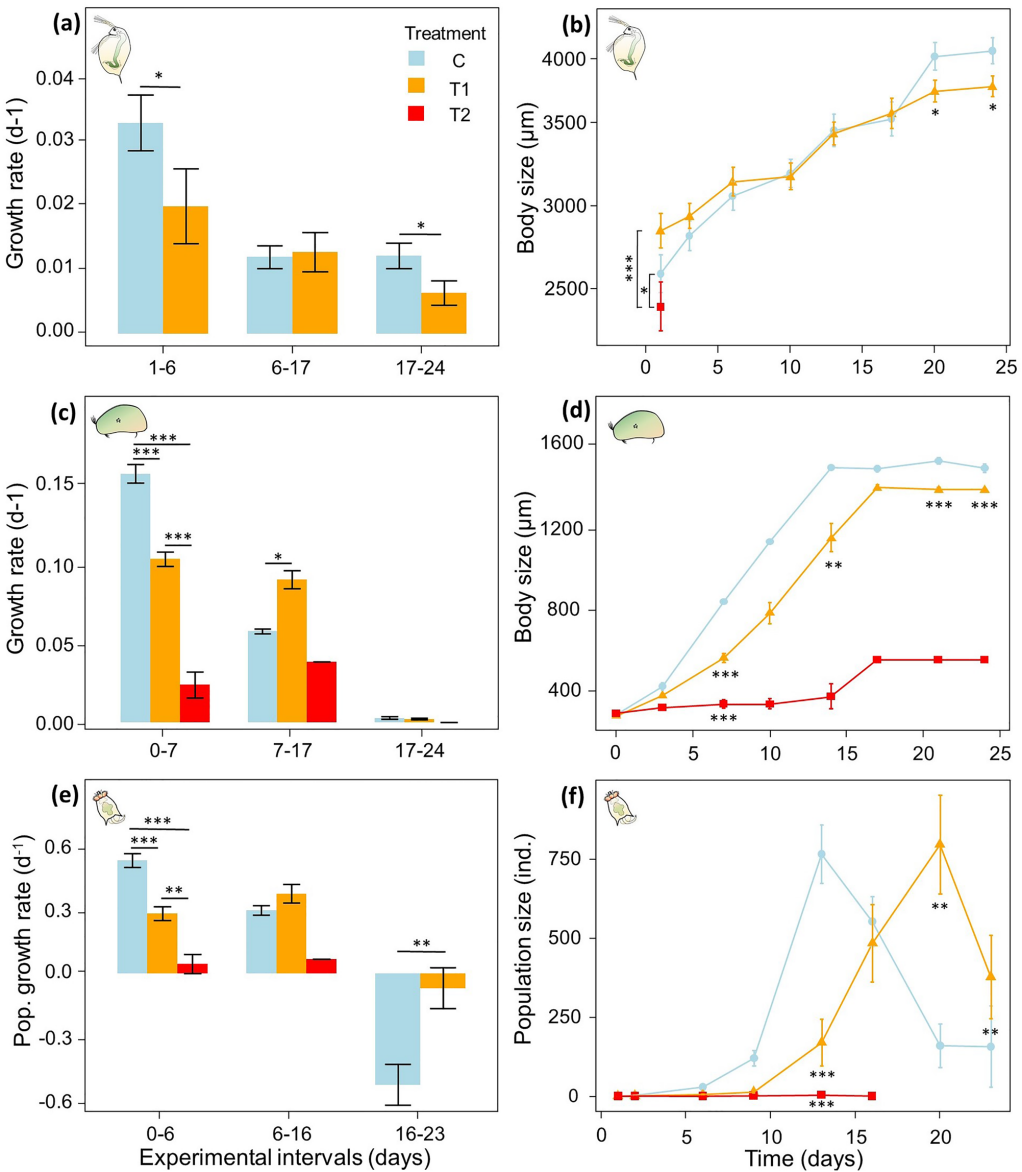
Life history responses of three freshwater zooplankton species subjected to a control (C = 1,520 ppm; blue circle), an elevated (T1 = 25,609 ppm; orange triangle) and an extreme (T2 = 83,201 ppm; red square) pCO_2_ treatment including water flea (*D. magna*) (**a**) somatic growth rate per experimental interval (day 1–6 = int. 1, 6–17 = int. 2, 17–24 = int. 3), (**b**) body size in time in µm; seed shrimp (*H. incongruens*) (**c**) somatic growth rate per experimental interval (day 0–7 = int. 1, 7–17 = int. 2, 17–24 = int. 3), (**d**) body size in time in µm; and rotifer (*B. calyciflorus*) (**e**) population growth rate per experimental interval (day 0–6 = int. 1, 6–16 = int. 2, 16–23 = int. 3) and (**f**) population size in time. Error bars represent standard errors. Significant differences are indicated with asterisks: *p < 0.05, **p < 0.01, ***p < 0.001.

T1 reduced somatic growth rates in the water flea by 33% during the first 6 days (LMM, χ^2^_1_ = 5.235, *p* = 0.022) and by 45% in the last 8 days of the experiment (LMM, χ^2^_1_ = 4.310, *p* = 0.038), while no effects were found in the second interval (day 6–17; LMM, χ^2^_1_ = 0.022, *p* = 0.882; Fig. 2a; Table S2, Appendix 1). After one day of exposure to T2, water fleas were 8% smaller than in controls (LMM day 1, χ^2^_2_ = 16.468, *p* < 0.001; Fig. 2b). Afterwards body size data are deficient in this treatment given that the individuals died. Exposure to T1 reduced water flea body size by 5% after 24 days (LMM, χ^2^_1_ = 4.654, *p* = 0.031). However, this effect differed between water flea clones (LMM, Treatment × clone, χ^2^_2_ = 8.172, *p* = 0.017; Figure S1, Appendix 2). The treatment effect on body size also varied over time, both in the water flea (LMM, Treatment × Time, χ^2^_1_ = 9.893, *p* = 0.002) as well as in the seed shrimp (LMM, Treatment × Time, χ^2^_1_ = 11.920, *p* < 0.001). In the water flea, the negative effects of pCO_2_ only appeared after 20 days (LMM, χ^2^_1_ = 4.057, *p* = 0.044), while in the seed shrimp, body size is already reduced by 33% after 7 days (Anova, F_2_ = 216.7, *p* < 0.001; Fig. 2d). In this species, growth rate over the first 7 days was 34% lower in T1 and 85% lower in T2 compared to controls (Anova, F_2_ = 112.4, *p* < 0.001; Fig. 2c). Growth rate in T1 caught up in the second interval with 36% higher growth rates for seed shrimp compared to controls from day 7 to 17 (Mann Whitney U: W = 40, *p* = 0.003) and similar growth rates in the final 8 days (t test, t = 0.611, df = 12, *p* = 0.552). By the end of the experiment, T1 reduced seed shrimp body size by 7% (t-test, t = 4.922, df = 9, *p* < 0.001). In T2, body size was reduced by 76% after 14 days (Anova, F_13_ = 170.7, *p* < 0.001). This effect was no longer significant after day 14 due to high mortality; however the trend was maintained. T1 also delayed maturation in this species by 7 days (no variation) and decreased body size at maturity by 8% (Mann Whitney U, W = 40, *p* = 0.002). In T2, none of the animals managed to mature during the experiment.

The effect of elevated pCO_2_ on rotifer population growth rates and population sizes also changed over the course of the experiment (Table S2). pCO_2_ reduced clonal population growth rates by 47% in T1 and 91% in T2 during the first 6 days of exposure (Anova, F_2_ = 33.68, *p* < 0.001; Fig. 2e). During the next 10 days, growth rates were similar in T1 and control treatments and populations died in T2 (T-test, t = −1.606, df = 15, *p* = 0.129). In the final 8 days, both the controls and the T1 populations experienced negative growth rates but this effect was weaker in T1 than in the control (T-test, t = −3.319, df = 21, *p* = 0.003). Accordingly, both elevated pCO_2_ scenarios resulted in a reduced population size of 78% in T1 and 99% in T2 on day 13 (Anova, F_2_ = 31.9, *p* < 0.001; Fig. 2f). However, the T1 rotifers caught up by day 20, resulting in an 80% higher population size than controls (T-test, t = −3.733, df = 14, *p* = 0.002) and by the end of the experiment it was 58% higher than in controls (T-test, t = −3.185, df = 21, *p* = 0.005). Overall, this indicates that a similar population peak (Anova, F_2_ = 49.019, *p* < 0.001; Tukey post-hoc, *p* = 0.515) and subsequent crash is reached in the controls and under T1. However, there is a trend toward a delayed peak population by approximately 3 days in T1 (GLM, LR χ^2^_1_ = 3.212, *p* = 0.073). An average population size of 891 ± 352 s.d. and 729 ± 540 individuals was reached after 14 ± 3 and 17 ± 6 days in control and T1, respectively.

**Figure 3:**
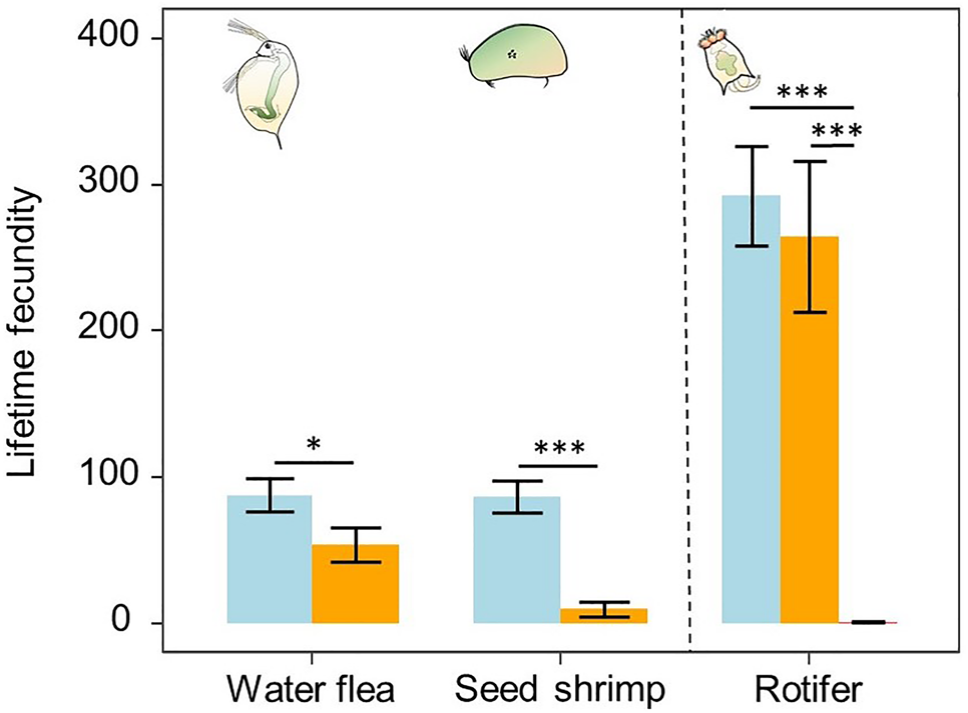
Lifetime fecundity responses of three freshwater zooplankton species subjected to a control (C = 1,520 ppm), an elevated (T1 = 25,609 ppm) and an extreme (T2 = 83,201 ppm) pCO_2_ treatment. Water flea (*D. magna)* and seed shrimp (*H. incongruens*) responses represent total number of neonates per female. Rotifer (*B. calyciflorus*) response represents total number of females with eggs per population. Error bars represent standard errors. Significant differences are indicated with asterisks: *p < 0.05, **p < 0.01, ***p < 0.001.

The water flea and the seed shrimp did not manage to reproduce under T2 conditions. Under T1, effects differed between species. In the water flea, T1 did not affect mean daily fecundity (LMM, χ^2^_1_ = 0.514, *p* = 0.473). However, it did reduce lifetime fecundity by 39% (GLMM, χ^2^_1_ = 4.658, *p* = 0.031; Fig. 3). In the seed shrimp, both mean daily fecundity (T-test, t_11_ = 6.451, *p* < 0.001) and lifetime fecundity were reduced by 89% (T-test, t_11_ = 6.451, *p* < 0.001). In the rotifer, mean daily fecundity of the population was 87% lower in T2 but unaffected in T1 (ANOVA, F_2_ = 41.922; *p* < 0.001). Lifetime fecundity was reduced by almost 100% in T2 (Tukey posthoc, *p* < 0.001), but not in T1 (Tukey posthoc *p* = 0.499).

## Discussion

The extent to which organisms from freshwater ponds and lakes will be affected by increasing pCO_2_ remains poorly understood. Some studies suggest that primary consumers will be indirectly affected since increased pCO_2_ can alter the nutritional quality of the primary producers on which they feed^16,19^. However, potential direct effects of exposure to pCO_2_ on life history, independent of changes in their food, remain unclear. Here, we exposed three model species representing three main functional groups of primary consumers – a water flea, a seed shrimp and a rotifer – to both elevated and extreme pCO_2_ levels. We excluded indirect effects of pCO_2_ and found that both an elevated and an extreme pCO_2_ level resulted in strong life history responses in all three tested species. Since responses were taxon specific, pCO_2_ driven shifts in the composition of freshwater zooplankton communities are likely.

Survival in all three study species was only reduced under extreme pCO_2_ (~ 83,000 ppm) conditions which resulted in a pH of 6.33 (± 0.08 s. d.). After only three days, mortalities varied between 100% in the water flea, 23% in the seed shrimp and 31% in the rotifer. Current literature shows that weak acidification (pH 5.5–7.0) in freshwater^5^, associated with high pCO_2_, can result in direct mortality in fish^33^, snails^34^, bivalves^35^ and frog larvae^36^. However, mortality induced by pCO_2_ has not been reported in freshwater zooplankton. Here, we show that this is possible but only under extreme concentrations that are currently highly uncommon (Table S1, Appendix 1) and unlikely to become common in natural ecosystems. Problems with calcification under low pH^29,37^ and elevated pCO_2_^22,23^ could explain the observed mortality in both the water flea and the seed shrimp since they have a calcified carapace, but this is speculative.

Weiss et al. (2018)^6^ concluded that pCO_2_ and not pH was responsible for the decreased expression of morphological anti-predator defenses in two *Daphnia* species, since this response was not observed when the same pH reduction was achieved using another acid. High levels of CO_2_ in the water have been shown to induce hypercapnia in crustaceans which leads to metabolic depression and decreased respiration^38,39^. These physiological changes may impact life history traits and can potentially result in mortality. For instance, higher mortality and delayed growth after exposure to elevated pCO_2_ in larvae of a marine copepod was attributed to increased energetic costs to maintain metabolic homeostasis^40^.

Although we did not find an effect of ~ 25,500 ppm pCO_2_ on mortality in any of the studied model species, several other key life history traits were strongly affected. For instance, growth in both the water flea and the seed shrimp was reduced under elevated pCO_2_. Such an effect was not detected in a previous study during which *Daphnia longicephala* and *D. pulex* were exposed to 16,000 ppm of pCO_2_^6^. Most likely, exposure time in that study was too short (i.e. 7 days vs. 24 days in our experiment) to induce or detect any life history responses. Reduced growth can result from a slower metabolism and increased allocation of energy towards metabolic homeostasis and defense mechanisms against acidosis^23,39,40^. If elevated pCO_2_ can lead to a reduction in body size, such effects might be exacerbated by higher temperatures which have been linked to smaller body sizes in cold blooded animals^41^, but this remains to be demonstrated. In the field, smaller body sizes might also be stimulated by indirect effects of pCO_2_ on phytoplankton. When a closely related water flea, *D. pulicaria*, was fed algae that had been previously cultured under elevated pCO_2_, water fleas also showed reduced growth^19^. However, this explanation is not valid for our study given that we fed dead algae grown under normal pCO_2_ conditions. As such, our study provides evidence for direct effects of elevated pCO_2_ on growth, which have not been demonstrated earlier in freshwater zooplankton.

Increased pCO_2_ also resulted in developmental delays. In the seed shrimp, there was an initial decrease in somatic growth of 34%, which was compensated after one week. This type of compensatory growth has been observed in other freshwater invertebrates in response to stressors^42^. However, the pCO_2_ exposed seed shrimp showed a delay in maturation, resulting in an 89% decrease in lifetime fecundity measured as the total no. of neonates. This indicates that short term acclimation to higher pCO_2_ comes with fitness costs in terms of lower per capita reproduction. In the rotifer, we found a trend of delayed population growth under elevated pCO_2_ levels, but it was not significant. Although such developmental delays may seem subtle, they could still impact the demography of natural populations. In short lived temporary pond systems, for instance, delayed maturation can prevent successful recruitment when populations fail to reproduce before the pond dries out^43^. In permanent systems, delays in population growth can disturb successional plankton dynamics^44^.

Similar as in the seed shrimp, the total reproductive output of the water flea was also reduced by an elevated pCO_2_ of ~ 25,000 ppm. However, this was not the result of delayed maturation since water fleas were already mature at the start of the experiment. A lower number of offspring under an elevated pCO_2_ of 7,000 ppm was also recently observed in *D. pulex*, by Pötter et al. (2021)^24^, however only in presence of predator cues. Here, we show that elevated pCO_2_ reduces the total number of offspring in different clonal lineages of *D. magna* exposed for 24 days. This is in accordance with findings of Parra et al. (2016)^45^ where a single *D. magna* clone was exposed to a CO_2_-induced acidification from pH 8.7 to 7.0. However, remarkably this study did not report actual pCO_2_ values which limits conclusions and prevents direct comparison with our results.

While water fleas were most sensitive in this study, it should be noted that individuals of *D. magna* were mature at the start of the exposure while the other species were juvenile. However, it is reasonable to assume that juvenile water fleas are even more sensitive than adults since previous studies have shown that early developmental stages are typically more sensitive to environmental stress such as elevated pCO_2_^40,46,47^.

If the responses in these three model species turn out to be representative of the broader taxonomical groups they represent, direct effects of pCO_2_ might contribute to functional shifts in freshwater communities. For instance, if larger water flea species are indeed systematically more sensitive to elevated pCO_2_ – as suggested by higher mortality in this study—control of algal blooms by these filter feeders may be compromised. This, in turn, could lead to smaller water flea species or rotifers increasing in importance as pelagic filter feeders. However, this remains to be confirmed by exposing communities of competitors to different pCO_2_ treatments.

The experiment performed here ran across a longer and more ecologically relevant time-scale than some earlier experiments with zooplankton (e.g.^6^) and therefore provides a more realistic test of the likely responses of individuals. For instance, individuals may be able to acclimate to an environment that frequently experiences elevated pCO_2_. Such acclimation mechanisms could include an improved capacity of CO_2_ buffering, transport and exchange as observed in deep-sea fish and invertebrates adapted to fluctuating pCO_2_ environments^48^. We found a few indications for acclimation in the accelerated growth of the seed shrimp and the compensation of an initial reduction in population growth in the rotifer. As acclimation via phenotypic plasticity could be important in the field, we must be careful not to overestimate effects of pCO_2_ based on exposure experiments. Also, genetic diversity and associated differential sensitivity of specific genotypes may impact the response of natural populations. While the tested rotifer and seed shrimp were homozygous laboratory populations, several different genetic water flea genotypes were used. Some genotypes were more sensitive and died under ~ 25,500 ppm pCO_2_, some survived but at the cost of a reduced body size while others were relatively unaffected. This suggests adaptive potential to cope with an environment characterized by elevated pCO_2_ and weak acidification, at least in the water flea, but likely in all tested species.

We used natural poorly buffered pond water, of which the physicochemical properties cannot be adequately reconstructed in the laboratory starting from demineralized water. While this has merits with regard to realism, it also implies that the studied responses may not be representative for the full range of natural pond and lake conditions. In well buffered systems, pCO_2_ effects will be weaker. A logical next step would be to investigate if effects of elevated pCO_2_ can be effectively mitigated via the buffering capacity of the water and to test whether a similar pH drop generated by other proton donors than carbonic acid would lead to different life history responses.

Overall, this study serves as a proof of principle that pCO_2_ can have direct effects on representatives of different functional groups of primary consumers in freshwater. These effects can be substantial at concentrations that have already been measured in the field and could become more common for many freshwater environments in the near future. How common such conditions may become is still unknown. The reason being that pCO_2_ in freshwater results from a combination of physiochemical conditions such as atmospheric CO_2_, temperature and different biological processes such as photosynthesis and respiration e.g. via decomposition of allochthonous organic matter^14^. As such there is a need for quantitative models to assess how changes in atmospheric CO_2_ will be reflected in pCO_2_ in different freshwater systems. Nevertheless, we show that the life history of freshwater zooplankton is sensitive to the physicochemical properties of CO_2_. While this insight is valuable, in natural environments direct and indirect pCO_2_ effects (e.g. via modulation of food and pH changes) operate simultaneously, complicating the ultimate response. At the very least the current observations confirm that predictive models for the performance of aquatic organisms under different climates should not simply focus on indirect effects of CO_2_ but also integrate direct effects.

## Methods

### Animal culture and medium

Five different clonal lineages of the water flea *Daphnia magna* were sampled from two ponds on agricultural land in Belgium (Vleteren: 50°55′06.7″ N, 2°43′27.0″ E and De Haan 51°13′53.8″ N, 3°01′49.2″). They were cultured separately in 210 ml glass jars under optimized laboratory conditions (20 ± 1 °C, 14:10 h light:dark cycle). Seed shrimp and rotifer resting eggs were obtained from a commercial supplier (MicroBioTests Inc., *H. incongruens* strain MBT/1999/10, product code TB36; *B. calyciflorus*, product code TK21, Belgium) and represent laboratory cultured, single clonal lineages. More details on animal culture are reported in the online supplementary methods (Appendix 3).

Natural pond water was used as medium both in animal cultures and the experiment. It was extracted from a Belgian region (50°59′00.92″ N, 5°19′55.85″ E, Zonhoven) with soft, poorly buffered water (Alkalinity 3–8°d; pH 6.5–8.5) which is likely to be susceptible to acidification under elevated pCO_2_. More information on medium and mineral composition is reported in the online supplementary information (Appendix 3; Table S3, Appendix 1).

### Experimental set-up

Organisms were exposed to three pCO_2_ treatments, an ambient control (C; 1,520 ppm ± 702 SD), an elevated (T1; 25,609 ppm ± 4,541 SD) and an extreme pCO_2_ level (T2; 83,201 ppm ± 15,533 SD). The control pCO_2_ level represents the current global mean that is measured in lentic freshwaters considering most ponds and lakes are already supersaturated^10,12^. The T1 level is currently only observed in more extreme cases^11^. However, it reflects a pCO_2_ level that could be encountered more commonly in the field in the future. The T2 treatment represents an extreme test of the tolerance limits of extant species. These treatments are a necessary simplification of reality since pCO_2_ can experience strong fluctuations in ponds and lakes. An overview of freshwater pCO_2_ concentrations from literature can be found in Table S1 (Appendix 1).

The elevated pCO_2_ concentrations were manipulated in the water by injecting pure CO_2_ (99.998% pure, ALPHAGAZ CO2 SFC * B50-N48, Airliquide, Belgium) from gas cylinders into the water (cf.^49^) at a constant flowrate, using a high-pressure regulator (HBS 200–10.2,5; AirLiquide, Belgium) and a flow controller (Sho-rate model 1350G, Brooks Instruments, USA). In the control treatment, ambient air was supplied at a similar rate as the CO_2_ to ensure equal perturbation levels across all containers. Water of all experimental containers (including control) were also injected with ambient air to keep the water oxygenated. A relatively constant pCO_2_ was ensured by continuously monitoring pH and kept between a range of ~ 20,000–30,000 ppm (pH 6.9–6.7) for T1 and ~ 70,000–120,000 ppm (pH 6.4–6.1) for T2 (Figure S2, Appendix 2).

Each treatment included 13 replicate 210 mL glass jars per species, resulting in a total of 117 experimental units. Per replicate, one mature water flea (8–11 days old) was inoculated in a jar containing aerated pond water. The five clonal lineages were distributed evenly over the experimental conditions so that each condition had the same number of replicates per clone*.* Seed shrimp replicates each contained one newly hatched (< 24 h old) individual. An autoclaved pebble was provided in each jar as a substrate for oviposition. Rotifer replicates contained one newly hatched individual (< 24 h old) to start a clonal population. For the water flea and the seed shrimp, juveniles were counted and removed. Since these rotifers only have a lifespan of 4–7 days at 20 °C^50^, clonal rotifer populations were allowed to grow during the entire experiment. The experimental containers were kept under standardized laboratory conditions in an incubator (20 ± 1 °C; 14:10 h light:dark cycle; 6000 K warm white LEDs, ± 3700 lx). The animals were fed ad libitum with a solution of dead green algae (*Acutodesmus obliquus*, 100 × 10^6^ cells/ml) reconstituted from thawed aliquots stored at −20 °C.

Throughout the experiment, standard water quality variables were measured (Table 1). pCO_2_ and DIC was calculated using pH, temperature and alkalinity measurements according to Fasching et al. (2014)^51^ (formulas in Appendix 4; variability in Figure S2, Appendix 2). The CO2SYS program^52^ was also used to calculate pCO_2_ for reproducibility purposes, which resulted in similar values as reported here (Table S4, Appendix 1).

**Table 1:** Means and standard deviations (± s.d.) of measured water quality variables and calculated pCO_2_ and DIC concentrations (according to Fasching et al., 2014) during the experiment. Alkalinity was determined via sulphuric acid titration using a digital titrator (Hach, USA) at the start and in week two, while conductivity (at the end), temperature and pH (twice a week) was measured using different probes (Orion™ ROSS Ultra™ pH/ATC Triode™, Thermo Scientific, USA; Cond 330i, WTW, Germany). Treatments include ambient control (C), elevated (T1) and extreme (T2) pCO_2_.

Variables	C	T1	T2
Temperature (°C)	20.5 ± 0.5	19.9 ± 0.5	20.3 ± 0.5
Conductivity (µS/cm)	20.3 ± 0.5	538.5 ± 15.7	526.2 ± 22.0
Alkalinity (µmol/L)	2,800 ± 136	2,858 ± 235	2,860 ± 266
pH	8.1 ± 0.21	6.84 ± 0.08	6.33 ± 0.08
pCO_2_ (ppm)	1,520 ± 702	25,609 ± 4,541	83,201 ± 15,533
pCO_2_ (µatm)	1,510 ± 707	25,628 ± 4,575	83,263 ± 15,648
DIC (µmol/L)	2,841.10 ± 35.00	3,863.52 ± 177.80	6,088.89 ± 586.02

### Measured responses

Each experimental replicate was monitored at fixed intervals over 24 days (water flea: 3 × /week, seed shrimp/rotifer: 2 × /week) to determine life history traits and mortality (more details in Table S5, Appendix 1). As water fleas and seed shrimps were kept individually, mortality reflects individual survival. In the short-lived rotifers, mortality was assessed as survival of the clonal population in each jar.

Body size of water fleas (from eye to spine base) and seed shrimp (longest straight line along the longitudinal axis) was measured up to the nearest µm under a stereomicroscope. Somatic growth rate was calculated for each of three intervals during the experiment. Mean daily fecundity was calculated as the average number of neonates per female per day in the water flea (released from brood chamber) and seed shrimp (hatched from eggs). The cumulative number of neonates produced per female at the end was calculated as a measure of lifetime fecundity. Seed shrimp age and size at maturity were determined from the moment the first eggs were found in the jars.

Population size of the rotifers was determined using a stereomicroscope, a Sedwick-Rafter counting chamber and Lugol’s solution for staining. Population growth rate was calculated as the intrinsic rate of population increase r = ln(F_2_) − ln(F_1_)/t_2_ − t_1_^53^ for each of three experimental intervals. The maximum population size of each clonal population and the timepoint at max. pop. size were also calculated. Mean daily fecundity was determined as the average number of individuals with eggs per day as a proxy for the fecundity of the clonal population^50^. The cumulative number of females with eggs at the end was determined as a measure of lifetime fecundity.

### Statistical analysis

All analyses were performed in R Studio version 1.0.143 (R version 3.6.1). The survival probability of each model species under the different treatments was estimated using Kaplan–Meier survival curves (ggsurvplot, *ggfortify* package). Differences in survival probability between treatments and clones and between species within treatments were tested using log rank tests (survdiff, *survival* package) and pairwise comparisons with Holm corrections (pairwise_survdiff, *survminer* package).

To test for the overall impact of the pCO_2_ treatments on water flea growth rate and body size, linear mixed models were used (LMM, lmer, *lme4* package). Treatment (C, T1, T2), time and their interaction were included as fixed predictors and the identity of the experimental jars (ID) as a random factor to correct for repeated measurements on the same individual. Clone and a treatment × clone interaction were also included to test for differences in the overall sensitivity of clones, or differences in the responses of clones to the treatments, respectively. To investigate the effect of the treatments at specific time points, separate LMMs were constructed for growth rate for each of three intervals (int. 1, 2, 3), body size on day one (since this is the only data point in T2) and body size for four time points (day 6, 13, 20, 24). Treatment was included as a fixed predictor and clonal identity as a random factor to correct for the dependency of measurements within the same clonal lineage. LMMs (lmer, *lme4* package) were constructed to investigate the effect of the treatments on water flea mean daily fecundity and lifetime fecundity, including treatment as fixed and clone as random factor.

LMMs were constructed to investigate the overall effect of the pCO_2_ treatments on seed shrimp growth rate and body size and rotifer population growth rate and size. In each model, ID was included as a random factor to account for repeated measurements. Analysis of variance (ANOVA, aov, *car* package) tests were used to test for the effect of the treatments at specific time points (growth rate: int. 1, 2, 3; body size: day 7, 14, 21, 24; population growth rate: int. 1, 2, 3; population size: day 6, 13, 20, 23) and for the effect on mean daily fecundity, lifetime fecundity, seed shrimp size at maturity and rotifer maximum population size. There was no variation in the age at maturity so no data analysis could be performed. The maximum population size timepoint was analyzed using a generalized linear model with a Poisson error distribution (GLMs, glm, *stats* package). The GLM was tested and corrected for overdispersion with the quasibinomial distribution.

Two out of the five inoculated water flea clones did not survive long enough in the T1 treatment to gather sufficient life history data. Therefore, these were excluded from analyses. In case of sometimes very high mortalities in T2, insufficient individuals remained for statistical analysis of certain life history traits. In that case, the T2 treatment was removed and t tests were used instead to test for differences between C and T1 (t-test, *stats* package). Normality of residuals and homogeneity of variances was verified using Shapiro–Wilk and Levene’s tests, respectively, for all linear models when applicable. In case of non-normal distribution of residuals, log transformation or non-parametric equivalents were used (Kruskall Wallis and Mann Witney U tests, *stats* package). Tukey post-hoc tests were performed (glht, *multcomp* package). Significance was always interpreted at *p* < 0.05.

## Supplementary Information

Supplementary Information.

## Data Availability

The datasets generated and/or analysed during the current study are available in the FigShare repository, 10.6084/m9.figshare.14885142.v1 .
