# Supplementary material for: Direct effects of elevated dissolved CO2 can alter the life history of freshwater zooplankton
Source: Sci Rep. 2022 Apr 12;12:6134. doi: 10.1038/s41598-022-10094-2 (PMC9005601; doi:10.1038/s41598-022-10094-2)
Supplement: Supplementary file 1 — Supplementary Information. [file 41598_2022_10094_MOESM1_ESM.docx]

**Direct effects of elevated dissolved CO_2_ can alter the life history of freshwater zooplankton**

**Lana Ramaekers^1, 2*^, Tom Pinceel^1, 2, 3^, Luc Brendonck^2, 4^ and Bram Vanschoenwinkel^1, 3^**

^1^Community Ecology Laboratory, Department of Biology, Vrije Universiteit Brussel (VUB), Pleinlaan 2, 1050 Brussels, Belgium

^2^Animal Ecology, Global Change and Sustainable Development, KU Leuven, Ch. Deberiotstraat 32, 3000 Leuven, Belgium

^3^Centre for Environmental Management, University of the Free State, P.O. Box 339, Bloemfontein, 9300 South Africa

^4^Water Research Group, Unit for Environmental Sciences and Management, North-West University, Private Bag X6001, Potchefstroom 2520, South Africa

*** Corresponding Author:** [lana.gitte.ramaekers@vub.be](mailto:lana.gitte.ramaekers@vub.be), [lana.ramaekers@kuleuven.be](mailto:lana.ramaekers@kuleuven.be)

**Other authors:** [tom.pinceel@kuleuven.be](mailto:tom.pinceel@kuleuven.be), [luc.brendonck@kuleuven.be](mailto:luc.brendonck@kuleuven.be), [bram.jasper.vanschoenwinkel@vub.be](mailto:bram.jasper.vanschoenwinkel@vub.be)

**Appendix 1: Supplementary tables**

**Table S1:** Overview of reported pCO_2_ concentrations from literature. Mean (or median) pCO_2_ with standard error (s.e.m.) or standard deviation (s.d.) and minimum - maximum range was reported when present. N represents number of measurements in different freshwater types.

| **Source** | **Freshwater type and region (no. lakes)** | **No. measurements (N)** | **Mean/median pCO_2_ (µatm)** | **Minimum pCO_2_ (µatm)** | **Maximum pCO_2_ (µatm)** |
| --- | --- | --- | --- | --- | --- |
| Cole et al. (1994) | Global lakes (37) | 390 | 801 + 67 s.e.m. | 10 | 4,128 |
| Cole et al. (1994) and citations herein | Full seasonal cycles of global lakes (69) | 2,395 | 680 | 5 | 7,991 |
| Cole et al. (1994) and citations herein | African lakes (59) | 79 | 2296 | 32 | 20,249 |
| Sobek and Tranvik (2005) | Global lakes (4902) | 12,898 | 1287 + 41 s.d. | 17 | 65,250 |
| Kortelainen et al. (2006) | Subset of Nordic Lakes Survey, Finland (177) | / | 1400 | 290 | 4,500 |
| Alin and Johnson (2007) and citations herein | Global large lakes (41) | / | 850 | 0 | 27,600 |
| Lazzarino et al. (2009) | Florida lakes, USA ( 948) | / | 3,550 | 0 | 81,000 |
| Balmer and Downing (2010) | Agriculturally eutrophic lakes, USA (131) | 3049 | 322 (median) | 0.1 | 40,392 |
| Raymond et al. (2013) | Global streams and rivers | 6,708 | 3,100 | / | / |
| Raymond et al. (2013) | Global lakes and reservoirs (7,939) | 20,632 | 839 | 340 | 1,906 |
| Raymond et al. (2013) | Global non-tropical lakes and reservoirs | / | 1,410 | / | / |
| Raymond et al. (2013) | Tropical lakes and reservoirs | / | 4,390 | / | / |
| Abril et al. (2015) | Global rivers and watersheds | 761 | 3,707 | 36 | 23,047 |
| Abril et al. (2015) | Leyre River, France | 92 | 4,429 | 901 | 23,047 |
| Abril et al. (2015) | Meuse River, Belgium | 50 | 2292 | 179 | 10,033 |
| Abril et al. (2015) | Amazon river, Brazil | 155 | 4,204 | 36 | 18,400 |
| Holgerson (2015) and citations herein | Global small temporary ponds (73) | / | 7,717 + 343 s.e.m. | 2,279 | 22,670 |
| Crawford et al. (2017) | Headwater streams, USA | >40,000 | / | 236 | 9,894 |
| Weiss et al. (2018 ) | German reservoirs (4) | / | 923.25 + 49.14 s.d. | / | / |

**Table S2**: Output of linear mixed models (LMM’s) testing the effects of treatment, time (experimental interval or day) and the treatment x time interaction on somatic (som.) growth rate and body size of the water flea D. magna and the seed shrimp H. incongruens and population (pop.) growth rate and population size of the rotifer B. calyciflorus. Seed shrimp body size and rotifer population size was split in two periods for analysis (seed shrimp: period 1 = day 3-10, period 2 = day 10-24; rotifer: period 1 = day 2-13, period 2 = day 13-23) because of high mortality in the extreme treatment (T2).The models of period 1 include all three treatments, in period 2 they only include control (C) and the elevated treatment (T1). For the water flea, T2 was not included in any model and also the effect of clone and the treatment x clone interaction was tested.

|  | | |  | **Water flea** | | |  | **Seed shrimp** | | | |  | **Rotifer** | | |  |
| --- | --- | --- | --- | --- | --- | --- | --- | --- | --- | --- | --- | --- | --- | --- | --- | --- |
| **LMM** | | |  | χ² | Df | *p* |  | | χ² | Df | *p* |  | χ² | Df | *p* |  |
| **Som./pop. growth rate** | | | |  |  |  |  | |  |  |  |  |  |  |  |  |
|  | Treatment | | | 6.667 | 1 | **0.010** |  | | 101.910 | 1 | **<0.001** |  | 8.920 | 1 | **0.003** |  |
|  | Time | | | 18.096 | 2 | **<0.001** |  | | 868.400 | 2 | **<0.001** |  | 181.813 | 2 | **<0.001** |  |
|  | Treatment x Time | | | 0.891 | 2 | 0.640 |  | | 11.920 | 2 | **<0.001** |  | 181.813 | 2 | **<0.001** |  |
|  | Clone | | | 4.034 | 2 | 0.133 |  | |  |  |  |  |  |  |  |  |
|  | Treatment x Clone | | | 2.646 | 2 | 0.266 |  | |  |  |  |  |  |  |  |  |
| **Body/ pop. size** | | | |  |  |  |  | |  |  |  |  |  |  |  |  |
|  | Treatment | | | 11.474 | 1 | **<0.001** |  | |  |  |  |  |  |  |  | |
|  | Time | | | 406.350 | 1 | **<0.001** |  | |  |  |  |  |  |  |  | |
|  | Treatment x Time | | | 9.893 | 1 | **0.002** |  | |  |  |  |  |  |  |  | |
|  | Clone | | | 31.881 | 2 | **<0.001** |  | |  |  |  |  |  |  |  | |
|  | Treatment x Clone | | | 8.172 | 2 | **0.017** |  | |  |  |  |  |  |  |  | |
|  | Period 1 | | |  |  |  |  | |  |  |  |  |  |  |  | |
|  |  | Treatment | |  |  |  |  | | 3.406 | 2 | 0.182 |  | 2.0649 | 2 | 0.356 | |
|  |  | Time | |  |  |  |  | | 503.206 | 1 | **<0.001** |  | 360.934 | 1 | **<0.001** | |
|  |  | Treatment x Time | |  |  |  |  | | 185.589 | 2 | **<0.001** |  | 62.198 | 2 | **<0.001** | |
|  | Period 2 | | |  |  |  |  | |  |  |  |  |  |  |  | |
|  |  | Treatment | |  |  |  |  | | 185.589 | 2 | **<0.001** |  | 39.138 | 1 | **<0.001** | |
|  |  | Time | |  |  |  |  | | 30.749 | 1 | **<0.001** |  | 65.213 | 1 | **<0.001** | |
|  |  | Treatment x Time | |  |  |  |  | | 15.187 | 1 | **<0.001** |  | 45.992 | 1 | **<0.001** | |

**Table S3:** Mineral composition of the pond water in the control (C) and elevated (T1) and extreme (T2) pCO_2_ treatment in our experiment. Elemental concentrations were measured with inductively coupled plasma mass spectrometry (ICP-MS; Agilent 7700x).

| **Component** | **Control** | **T1** | **T2** |
| --- | --- | --- | --- |
| **Na (mg/L)** | 79.00 | 83.79 | 71.68 |
| **Mg (mg/L)** | 4.32 | 4.55 | 4.22 |
| **Al (µg/L)** | 35.60 | 11.82 | 14.27 |
| **P (µg/L)** | 344.45 | 206.38 | 170.78 |
| **K (mg/L)** | 8.39 | 9.19 | 8.012 |
| **Ca (mg/L)** | 14.64 | 18.27 | 15.76 |
| **Cr (µg/L)** | 0.31 | 0.37 | 0.28 |
| **Mn (µg/L)** | 2.05 | 1.80 | 0.28 |
| **Fe (µg/L)** | 11.49 | 6.47 | 2.92 |
| **Co (µg/L)** | 0.13 | 0.11 | 0.10 |
| **Ni (µg/L)** | 0.95 | 0.93 | 0.92 |
| **Cu (µg/L)** | 11.27 | 5.48 | 10.18 |
| **Zn (µg/L)** | 12.05 | 11.59 | 29.37 |
| **Ga (µg/L)** | 1.95 | 1.69 | 1.80 |
| **As (µg/L)** | 1.20 | 1.27 | 1.18 |
| **Sr (µg/L)** | 100.98 | 121.13 | 112.00 |
| **Zr (µg/L)** | 1.93 | 3.31 | 2.95 |
| **Mo (µg/L)** | 0.78 | 0.82 | 0.70 |
| **^111^Cd (µg/L)** | 0.54 | 10.31 | 0.06 |
| **^114^Cd (µg/L)** | 0.55 | 10.90 | 0.05 |
| **In (µg/L)** | 0.02 | 0.01 | 0.01 |
| **Sn (µg/L)** | 0.29 | 0.11 | 0.12 |
| **Cs (µg/L)** | 0.04 | 0.05 | 0.04 |
| **Ba (µg/L)** | 14.09 | 12.28 | 12.89 |
| **Pb (µg/L)** | 0.59 | 0.39 | 0.55 |
| **U (µg/L)** | 0.16 | 0.12 | 0.06 |

**Table S4:** Comparison of pCO_2_ calculation (mean + standard deviation) according to Fasching et al., 2014 (**Appendix 4**) and the CO2SYS program (Pierrot et al., 2011) for the control (C), elevated (T1) and extreme (T2) pCO_2_ treatments in our experiment. Also included are the values for the different carbon ions (HCO_3_^-^, CO_3_^2-^ and CO_2_) and their relative percentages.

| **Variables** | **C** | **T1** | **T2** |
| --- | --- | --- | --- |
| **pCO_2_ (ppm)*** | 1,520 + 702 | 25,609 + 4,541 | 83,201 + 15,533 |
| **pCO_2_ (ppm)**** | 1,560 + 725 | 26,262 + 4,689 | 85,355 + 16,061 |
| **HCO_3_^-^ (µmol/kg)**** | 2,766 + 18.00 | 2,858 + 0.31 | 2,860 + 0.18 |
| **CO_3_^2-^ (µmol/kg)**** | 16.3 + 9.00 | 0.8 + 0.14 | 0.3 + 0.05 |
| **CO_2_ (µmol/kg)**** | 58 + 27 | 1,004 + 178 | 3,227 + 585 |
| **% HCO_3_**** | 97.37 | 73.99 | 46.99 |
| % **CO_3_^2-^**** | 0.57 | 0.02 | 0.004 |
| % **CO_2_**** | 2.06 | 25.98 | 53.01 |

*Calculated according to Fasching et al. 2014.

**Calculated according to the CO2SYS program.

**Table S5:** Overview of different life history traits with frequency and technique of measurement per species. Growth rate/population growth was calculated for 3 intervals (int.) over the period of the experiment

| **Study species** | **Life history trait** | **Frequency** | **Technique** |
| --- | --- | --- | --- |
| Water flea  *Daphnia magna* | Body size | 3x/week | Stereo-microscope |
|  | Growth rate | Int. 1: day 1-6  Int. 2: day 6-17  Int. 3: day 17-24 | Calculated from body size using formula:  ln(BS_1_)-ln(BS_0_)/(t_1_-t_0_) |
|  | Mean daily fecundity  Lifetime fecundity | 3x/week | Counting #neonates per female per clutch |
|  | Mortality | Daily | No observed movement |
| Seed shrimp  *Heterocypris incongruens* | Body size (BS) | 2x/week | Stereo-microscope |
|  | Growth rate (GR) | Int. 1: day 0-7  Int. 2: day 7-17  Int. 3: day 17-24 | Calculated from body size using formula:  ln(BS_1_)-ln(BS_0_)/(t_1_-t_0_) |
|  | Mean daily fecundity  Lifetime fecundity | 2x/week | Counting #neonates per female |
|  | Size at maturity  Age at maturity | Daily (starting from day 14) | Stereo-microscope, when first eggs laid |
|  | Mortality | Daily | No observed movement |
| Rotifer  *Brachionus calyciflorus* | Population size | 2x/week | Stereo-microscope/ Sedwick-Rafter counting chamber |
|  | Population growth | Int. 1: day 0-6  Int. 2: day 6-16  Int. 3: day 16-23 | Calculated from pop. size using formula:  ln(PS_1_)-ln(PS_0_)/t_1_-t_0_ |
|  | Mean daily fecundity  Lifetime fecundity | 2x/week | Stereo-microscope/ Sedwick-Rafter counting chamber, counting #females with eggs |
|  | Max. population size  Max. population size timepoint | / | Calculated from pop. size |
|  | Population mortality | Daily | No observed individuals |

Supplementary references (**Table S1**; rest in main manuscript)

- Cole, J. J., Caraco, N. F., Kling, G. W. and Kratz, T. K. Carbon supersaturation in the surface waters of lakes. *Science* **265,** 1568-1570 (1994).
- Kortelainen, P. et al. Sediment respiration and lake trophic state are important predictors of large CO_2_ evasion from small boreal lakes. *Global Change Biology* **12,** 1554-1567, <https://doi.org/10.1111/j.1365-2486.2006.01167.x> (2006).
- Alin, S. R. and Johnson, T. C. Carbon cycling in large lakes of the world: A synthesis of production, burial, and lake-atmosphere exchange estimates. *Global Biogeochemical Cycles* **21,** GB3002, <https://doi.org/10.1029/2006GB002881> (2007).
- Balmer, M. B. and Downing, J. A. Carbon dioxide concentrations in eutrophic lakes: undersaturation implies atmospheric uptake. *Inland Waters* **1,** 125-132, <https://doi.org/10.5268/IW-1.2.366> (2011).
- Holgerson, M. A. Drivers of carbon dioxide and methane supersaturation in small, temporary ponds. *Biogeochemistry* **124,** 305-318, <https://doi.org/10.1007/s10533-015-0099-y> (2015).
- Crawford, J. A., Stanley, E. H., Dornblaser, M. M. and Striegl, R. G. CO_2_ time series patterns in contrasting headwater streams of North America. *Aquatic Science* **79,** 473-486, <https://doi.org/10.1007/s00027-016-0511-2> (2017).

***
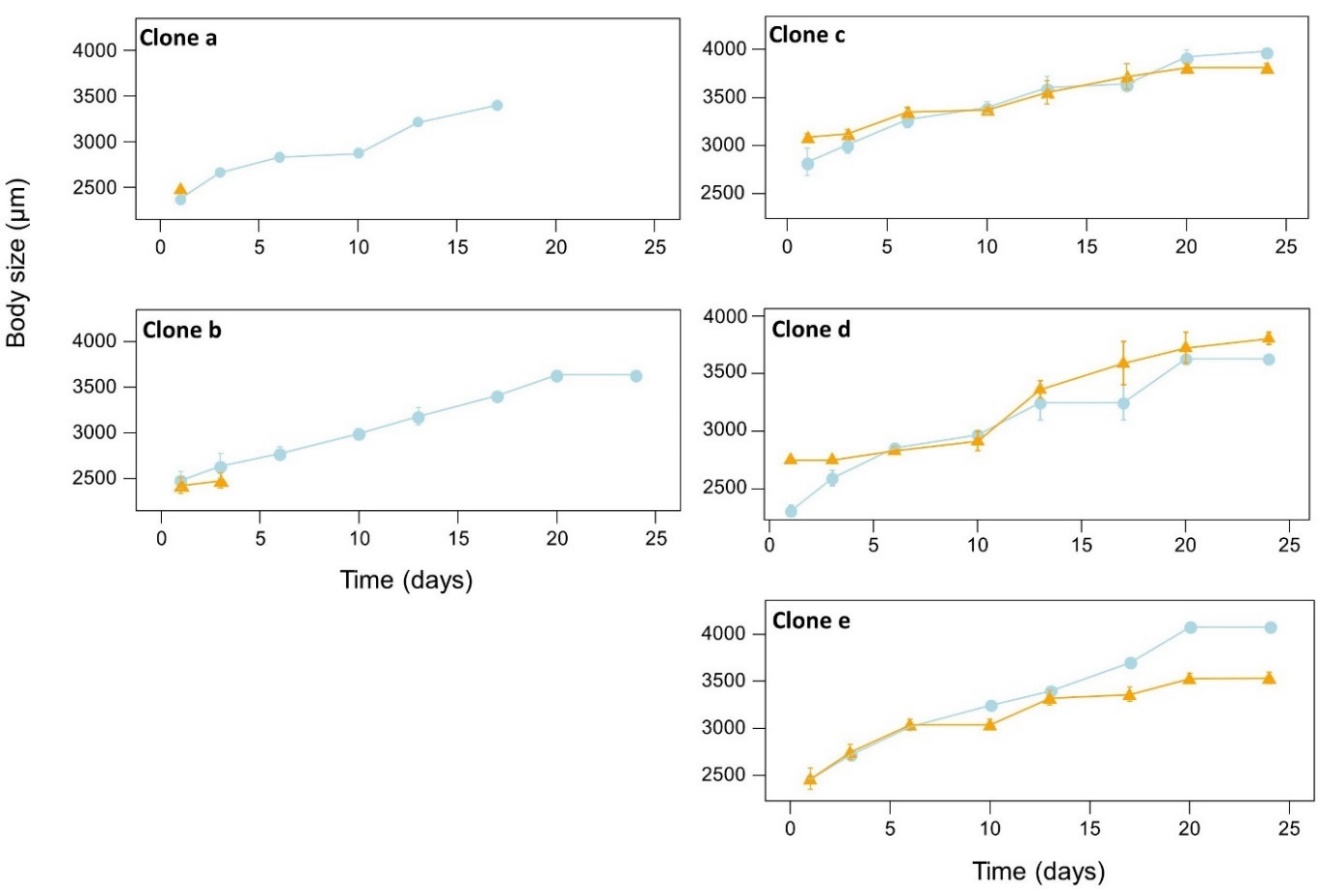
*Appendix 2: Supplementary figures**

**Figure S1**: Differential response in body size of *D. magna* water flea clones (a-e) subjected to a control (C = 1,520 ppm; ●) and an elevated (T1 = 25,609 ppm; ▲) pCO_2_ treatment. Error bars represent standard errors. Clone a and b were excluded from analysis since insufficient data was present in T1 due to high mortality.


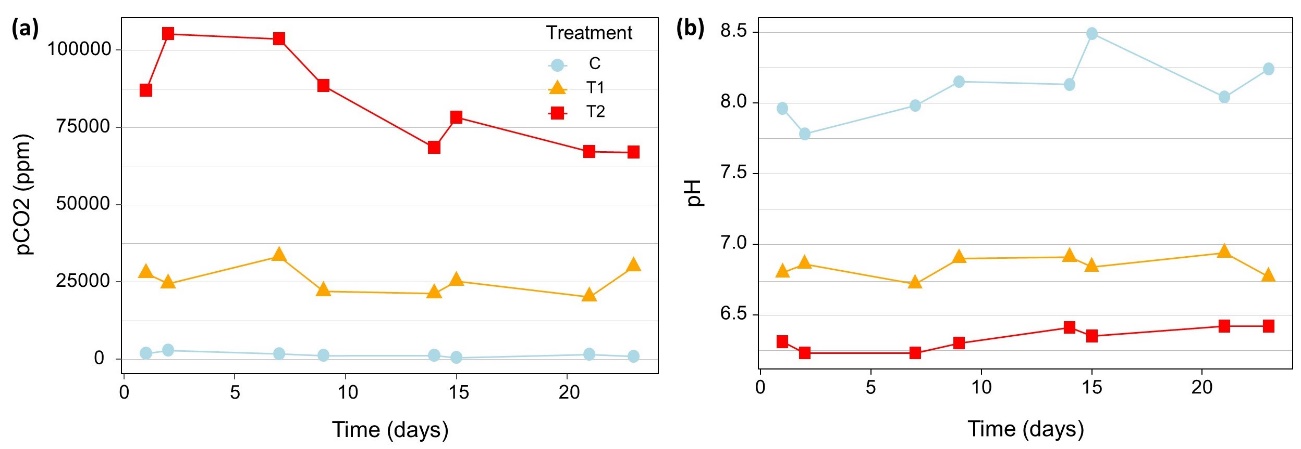


**Figure S2:** Time series of (a) calculated pCO_2_ (ppm) and (b) pH over the duration of the experiment. Treatments include a control (C = 1,520 ppm ; ●), an elevated (T1 = 25,609 ppm ; ▲) and an extreme (T2 = 83,201 ppm; ■) pCO_2_ treatment. Treatments were adjusted to stay within a range of ~ 20,000 – 30,000 ppm (pH 6.9 - 6.7) for T1 and ~ 70,000 – 120,000 ppm (pH 6.4 - 6.1) for T2.

**Appendix 3: Supplementary methods**

Animal culture and medium

Three freshwater model organisms were chosen to investigate the effects of elevated pCO_2_ on different functional groups of primary consumers. Functional groups differently affect the food web and the broader ecosystem because of differences in functional traits such as feeding mode and size. The water flea *Daphnia magna* (Crustacea: Cladocera; 1.5 - 6 mm, [1]) is a large efficient pelagic filter feeder of phytoplankton and a dominant competitor in many small pond ecosystems [2]. Ostracods or seed shrimp are generally more benthic opportunistic collector gatherers. However, several species such as *Heterocypris incongruens* (Crustacea: Ostracoda; 1.5-2.5 mm, [3]) can be found free swimming in the pelagic consuming phytoplankton [4]. Finally, the rotifer *Brachionus calyciflorus* (Rotifera; 0.18-0.57 mm, [5]), is a small pelagic filter feeder in a wide range of freshwater systems consuming individuals algal cells [6]. *Daphnia* species typically have a high calcium content associated with their exoskeleton compared to other water flea species [7]. While seed shrimp also have highly calcified valves [8], rotifers are less reliant on calcium since their lorica consists mainly of keratin-like proteins [9]. The three selected species have broad distributions and can alternate between asexual and sexual reproduction. Sexual reproduction is typically restricted to stressful conditions e.g. at the onset of winter, under food limitation or when water levels drop [10].

Water fleas were sampled from two ponds on agricultural land in the province of West-Flanders, Belgium (Vleteren: 50°55’06.7” N, 2°43’27.0” E and De Haan 51°13’53.8” N, 3°01’49.2”) in June 2018 and cultured in the lab for at least eight months under optimized laboratory conditions (20 + 1 °C, 16:8 h light:dark cycle). Five different clonal lineages were reared separately and individually in 210 ml jars in incubators under similar conditions (at 20 + 1 °C, 14:10 h light:dark cycle). They were fed frozen *Acutodesmus obliquus* green algae three times per week (500 µL, 100 x 10^6^ cells/ml) and 70% of the medium was refreshed twice a week. Offspring was removed from the jars to avoid crowding. Fourth brood neonates were isolated and reared to maturity (i.e. the moment when a first clutch of eggs is released in the brood pouch), under similar standardized conditions as described above, before treatment exposure. Seed shrimp and rotifer resting eggs were obtained from a commercial supplier (MicroBioTests Inc., *H. incongruens* strain MBT/1999/10, product code TB36; *B. calyciflorus*, product code TK21, Belgium). The seed shrimp originated from Ghent, Belgium and the rotifers from Florida, USA. Both are lab cultured and represent single clonal lineages. All resting eggs were inundated with EPA medium [11], which is demineralized water with 0.096 g/L NaHCO_3_, 0.06 g/L CaSO_4_-2H_2_O, 0.06 g/L MgSO_4_ and 0.004 g/L KCl, to reach a conductivity of 160 µS/cm, in petri dishes. The eggs were incubated at 25 °C and under permanent light conditions for 24 h for the rotifer and 52 h for the seed shrimp, before hatching. Hatchlings were transferred to 20 °C pond water for acclimatization and less than 24 h old when transferred to experimental conditions.

Natural pond water was used as medium to establish ecologically relevant conditions that mimic the complex water chemistry of ponds and a realistic level of buffering (i.e. the chemical ability to resist pH changes) that will lead to future pCO_2_ induced acidification. The water was extracted from a typical Western European fish pond in the ‘Midden-Limburg’ pond complex (Grote vijver, 50°59'00.92″ N, 5°19'55.85″ E, Zonhoven, Belgium) with soft, poorly buffered water in November 2018. These ponds have been used for intensive fish farming for many decades and therefore most suffer from eutrophication, with trophic states varying between mesotrophic and eutrophic [12,13]. The pond complex was chosen since it is representative for many similar ponds in the wider region including neighboring countries. The pond water was filtered three times through a 64 µm plankton net to exclude zooplankton. The mineral composition of the pond water is reported in **Table S3** (**Appendix 1**). Regular checks of the culture medium never revealed any metazoan zooplankton. It was stored in a 500 L container in a climate-controlled room at 20 °C, aerated and kept in darkness to prevent algal growth. Medium was refreshed (70 %) twice a week.

Supplementary references

1. Bledzki, L. A. and Rubak, J. J. *Freshwater Crustacean Zooplankton of Europe* (Springer International Publishing, 2016).
2. Ebert D. *Ecology, Epidemiology, and Evolution of Parasitism in Daphnia* Ch. 2 (National Center for Biotechnology Information, 2005).
3. Karanovic, I. *Recent Freshwater Ostracods of the World* (Springer-Verlag Berlin Heidelberg, 2012).
4. Meisch, C. *Freshwater Seed shrimp of Western and Central Europe*. (Spektrum Akademischer Verlag, 2000).
5. Kutikova, L. A. *A Guide to Tropical Freshwater Zooplankton: Identification, Ecology and Impact on Fisheries* (ed. C. H. Fernando) (Backhuys Publishers, 2002).
6. Segers, H. Global diversity of rotifers (Rotifera) in freshwater. *Hydrobiologia* **595,** 49-59, https://doi.org/ 10.1007/s10750-007-9003-7 (2008).
7. Waervagen, S. B., Rukke N. A. and Hessen D. O. Calcium content of crustacean zooplankton and its potential role in species distribution. *Freshwater Biology* **47,** 1866-1878 (2002).
8. Turpen, J. B. and Angell, R. W. Aspects of molting and calcification in the ostracod *Heterocypris*. *The Biological Bulletin* **140,** 331-338 (1971).
9. Bender, K. and Kleinow, W. Chemical properties of the lorica and related parts from the integument of *Brachionus plicatilis*. *Comparative Biochemistry and Physiology* **89B***,* 483-487 (1988).
10. Thorp, J. H. and Covich A. P. *Ecology and Classification of North American Freshwater Invertebrates.* Third Edition (Elsevier, 2010).
11. USEPA. *Methods for measuring the acute toxicity of effluents to freshwater and marine organisms.* Report EPA-821-R-02-012 (United States Environmental Protection Agency, 2002).
12. ANB. *Rapport 26: Instandhoudingsdoelstellingen voor speciale beschermingszones*. (Agentschap Natuur en Bos, 2011).
13. Lemmens, P. et al. How to maximally support local and regional biodiversity in applied conservation? Insight from pond management. *PLoS ONE* **8,** e7538, <https://doi.org/10.1371/journal.pone.0072538> (2013).

**Appendix 4: pCO_2_ calculation formulas**

$$xCO_{2}=\frac{pCO_{2}}{P_{field}}$$

$$pCO_{2}=\left( \frac{C{O_{2}}_{aq}}{K_{H}} \right)*{10}^{6}$$

$$CO_{2_{aq}}=\frac{DIC}{1+\frac{K_{1}}{\left[ H^{+} \right]}+\frac{K_{1}*K_{2}}{\left[ H^{+} \right]^{2}}}$$

$$DIC={(A}_{T}-\frac{K_{w}}{\left[ H^{+} \right]}+\left[ H^{+} \right])*(\frac{\left[ H^{+} \right]^{2}+K_{1}*\left[ H^{+} \right]+K_{1}*K_{2}}{K_{1}*\left[ H^{+} \right]+2*K_{1}*K_{2}})$$

xCO_2_ = partial pressure of CO_2_ as mole fraction (or volume) in ppmv (assuming ideal gas)

pCO_2_ = partial pressure of CO_2_ (µatm)

P_filed_ = air pressure (atm)

CO_2aq_ = concentration of dissolved CO^2^ (M/L)

K_H_ = Henry’s constant (adjusted for temperature after Weiss 1974)

DIC = dissolved inorganic carbon (M C/L)

K_1_ = Equilibrium constant for dissociation of H_2_CO_3_ (1^st^ acidity constant), adjusted for temperature (Stumm and Morgan, 1996)

K_2_ = Equilibrium constant or dissociation of HCO_3_^-^ (2^nd^ acidity constant), adjusted for temperature (Stumm and Morgan, 1996)

[H^+^] = proton concentration

A_T_ = total alkalinity (M/L)

K_w_ = ion product of water, adjusted for temperature (Stumm and Morgan, 1996)

Modified from Fasching et al. 2014.

Supplementary references

- Butler, J. N. *Carbon Dioxide Equilibria and Their Applications* (Lewis Publishers, 1991).
- Dickson, A.G., Sabine, C. L. and Christian, J. R. *Guide To Best Practices for Ocean CO_2_ Measurements* 191 (PICES Special Publication 3, 2007).
- Fasching, C., Behounek, B., Singer, G. A. and Battin, T. J. Microbial degradation of terrigenous dissolved organic matter and potential consequences for carbon cycling in brown-water streams. *Scientific reports* **4**, 1-7, <https://doi.org/10.1038/srep04981> (2014).
- Stumm, W. and Morgan, J. J *Aquatic Chemistry: Chemical Equilibria and Rates in Natural Waters* (John Wiley & Sons, 1996).
- Weiss, R. F. Carbon dioxide in water and sea water: The solubility of a nonideal gas. *Marine Chemistry* **2,** 203-21 (1974).
